# Renal Tubular Cell Mitochondrial Dysfunction Occurs Despite Preserved Renal Oxygen Delivery in Experimental Septic Acute Kidney Injury

**DOI:** 10.1097/CCM.0000000000002937

**Published:** 2018-03-14

**Authors:** Nishkantha Arulkumaran, Sean Pollen, Elisabetta Greco, Holly Courtneidge, Andrew M. Hall, Michael R. Duchen, Frederick W. K. Tam, Robert J. Unwin, Mervyn Singer

**Affiliations:** 1 Bloomsbury Institute of Intensive Care Medicine, Division of Medicine, University College London, London, United Kingdom.; 2 UCL Centre for Nephrology, Division of Medicine, Royal Free Campus and Hospital, University College London, London, United Kingdom.; 3 Imperial College Kidney and Transplant Institute, Hammersmith Hospital, Imperial College London, London, United Kingdom.; 4 Institute of Anatomy, University of Zurich, Winterthurerstrasse, Zurich, Switzerland.; 5 Department of Cell and Development Biology, University College London, London, United Kingdom.

**Keywords:** acute kidney injury, animal model, bioenergetics, sepsis

## Abstract

**Objective:**

To explain the paradigm of significant renal functional impairment despite preserved hemodynamics and histology in sepsis-induced acute kidney injury.

**Design:**

Prospective observational animal study.

**Setting:**

University research laboratory.

**Subjects:**

Male Wistar rats.

**Intervention:**

Using a fluid-resuscitated sublethal rat model of fecal peritonitis, changes in renal function were characterized in relation to global and renal hemodynamics, and histology at 6 and 24 hours (*n* = 6–10). Sham-operated animals were used as comparison (*n* = 8). Tubular cell mitochondrial function was assessed using multiphoton confocal imaging of live kidney slices incubated in septic serum.

**Measurements and Main Results:**

By 24 hours, serum creatinine was significantly elevated with a concurrent decrease in renal lactate clearance in septic animals compared with sham-operated and 6-hour septic animals. Renal uncoupling protein-2 was elevated in septic animals at 24 hours although tubular cell injury was minimal and mitochondrial ultrastructure in renal proximal tubular cells preserved. There was no significant change in global or renal hemodynamics and oxygen delivery/consumption between sham-operated and septic animals at both 6- and 24-hour timepoints. In the live kidney slice model, mitochondrial dysfunction was seen in proximal tubular epithelial cells incubated with septic serum with increased production of reactive oxygen species, and decreases in nicotinamide adenine dinucleotide and mitochondrial membrane potential. These effects were prevented by coincubation with the reactive oxygen species scavenger, 4-hydroxy-2,2,6,6-tetramethyl-piperidin-1-oxyl.

**Conclusions:**

Renal dysfunction in sepsis occurs independently of hemodynamic instability or structural damage. Mitochondrial dysfunction mediated by circulating mediators that induce local oxidative stress may represent an important pathophysiologic mechanism.

## Abstract 2

Supplemental Digital Content is available in the text.

Hemodynamic instability associated with sepsis and septic shock has led to the long-standing dogma that sepsis-induced acute kidney injury (AKI) is primarily a consequence of renal ischemia and ensuing acute tubular necrosis (1). However, multiple analyses in both patients and animal models of AKI fail to demonstrate significant histologic injury (2–4). Furthermore, measurement of renal blood flow (RBF) in septic patients with AKI revealed preserved or even elevated RBF (5–7), whereas experimental models reported no correlation between creatinine clearance and RBF (8, 9). Thus, AKI occurs notwithstanding adequate regional oxygen delivery and tissue oxygenation, at least at the macrovascular level. Even those patients with nonsevere sepsis without significant hemodynamic compromise nor requiring intensive care admission are at increased risk of AKI (10). The paradigm of significant functional impairment despite preserved global and renal hemodynamics and histology remains poorly understood. Whether the trigger for sepsis-induced AKI is intrarenal and/or mediated via the effect of circulating factors is also uncertain.

We previously proposed that organ dysfunction in sepsis is related to mitochondrial dysfunction (11). To investigate the role of mitochondrial dysfunction and the contribution of circulating factors to infection-associated renal dysfunction, we utilized a well-characterized, clinically relevant, fluid-resuscitated, nonlethal rat model of fecal peritonitis (12, 13). In addition, we performed ex vivo studies using multiphoton imaging of live kidney slices incubated in septic and control serum to assess, independent of any change in RBF or oxygen delivery, the effects of circulating factors associated with sepsis on renal tubular mitochondrial function.

## MATERIALS AND METHODS

### Animal Model of Fecal Peritonitis

**Figure 1.**
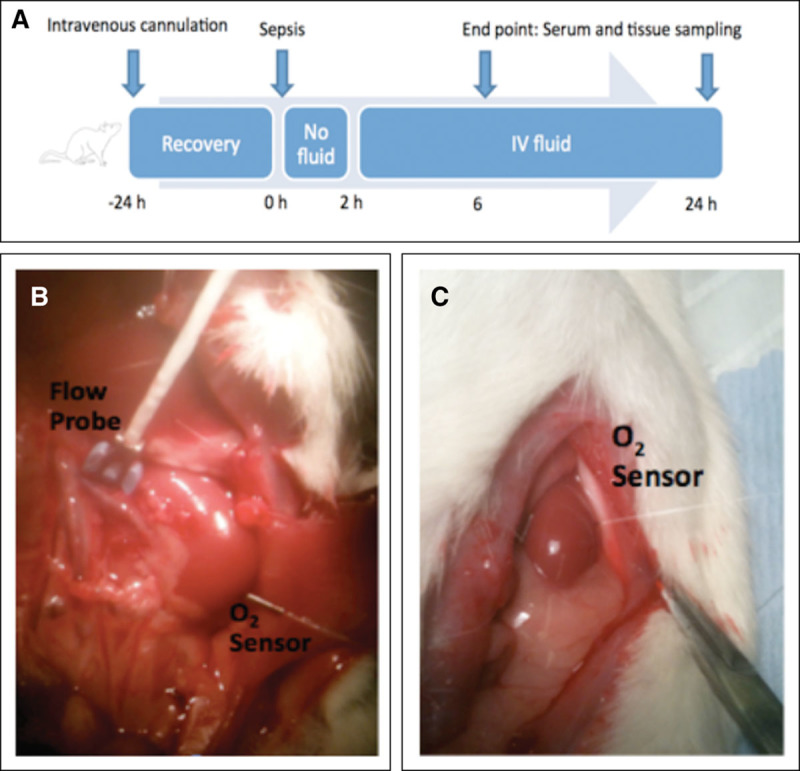
In vivo experimental set up. **A**, Schema of experimental set up. Animals have a tunneled central venous catheter inserted 24 hours prior to induction of sepsis. At 2 hours, IV fluid resuscitation is commenced. Animals are anesthetized and laparotomy performed at 6 or 24 hours for measurement of cardiac output, renal blood flow, renal cortical oxygen tension and renal vein oxygen tension, and renal tissue sampling. **B**, Flow probe measuring renal blood flow. Oxygen sensor inserted into lower pole of renal cortex. **C**, Oxygen sensor inserted into the mid-pole of the renal cortex.

Male Wistar rats (Charles River, Margate, United Kingdom) weighing 300–375 g were used throughout. All experiments were performed under a Home Office Project License (PPL 70/7029) and local UCL Ethics Committee approval. All experiments were performed in accordance with relevant guidelines and regulations. A similar model has been described in detail elsewhere (12–14) and the outline illustrated in **Figure 1**. However, in contrast to previous experiments, this model used human fecal slurry (13) to produce nonlethal sepsis with characteristics of rat fecal slurry peritonitis (12, 14).

The dose of slurry was selected to create a nonlethal model of sepsis, as the objective of this study was to investigate mechanisms of AKI in the absence of significant hemodynamic compromise. Laparotomy was performed under anesthesia at either 6- or 24-hour postinduction of sepsis, with echocardiography performed beforehand.

After laparotomy, a 22-gauge needle was used to puncture the renal capsule at the mid-pole. A fiberoptic optode (250 µm diameter) connected to an Oxylite monitoring system (Oxford Optronix, Didcot, Oxon, United Kingdom) allowed continuous tissue oxygen tension (tPo_2_) monitoring within the renal cortex (15) (**Fig. 1**). The left renal artery was isolated by careful blunt dissection. An ultrasonic flow probe (Transonic Systems, Ithaca, NY) of 1 mm diameter was placed around the left renal artery to measure RBF. Renal lactate clearance was calculated using the difference between renal vein and arterial lactate.

Further details are provided in the **supplemental data – Methods** (Supplemental Digital Content 1, http://links.lww.com/CCM/D123).

### Histology—Light Microscopy

Kidneys were fixed for 24–72 hours in formalin, transferred to 70% ethanol, and embedded in paraffin. Sections were then cut into 5 µm slices and mounted onto glass slides. Sections were examined by light microscopy (BX4, Olympus Optical, London, United Kingdom) at ×20 magnification. All sections were stained with Periodic acid-Schiff.

Apoptosis was identified by DNA fragments in situ using the terminal deoxyribonucleotidyl transferase-mediated biotin-16-dUTP nick-end labeling (TUNEL) assay (TACS TdT In Situ Apoptosis Detection Kit, R&D Systems, Abingdon, Oxford, United Kingdom).

### Histology—Electron Microscopy

As the instrumentation methodologies described above may potentially affect renal histology, samples for electron microscopy were taken in repeat experiments at 24 hours, where instrumentation was not performed. A kidney was obtained from septic and sham animals under anesthesia. Renal tissue was cut into sections of approximately 1 mm^3^ and placed immediately into glutaraldehyde fixative. The histopathology laboratory at the Royal Free Hospital, London, performed electron microscopy. Analysis was qualitative and focused on mitochondrial structure within proximal tubular epithelial cells (PTECs). Higher magnifications (×40,000) were used to evaluate changes within the mitochondria.

### Ex Vivo Assessment of Mitochondrial Function Using Confocal Microscopy

Using dyes or natural fluorophores, confocal microscopy allows detailed imaging of cellular physiologic processes in intact renal tissue sections. Multiphoton imaging uses a long wavelength excitation laser that permits greater tissue penetration compared with conventional single-laser confocal fluorescence microscopy. This technique can image live kidney slices in real-time in response to various insults or drugs. We previously reported the use of multiphoton imaging of freshly prepared rat kidney slices to investigate mitochondrial function in cells along the nephron in response to toxic stimuli, including chemical anoxia (16).

Further details are provided in the online data supplement (Supplemental Digital Content 1, http://links.lww.com/CCM/D123). In brief, the cationic lipophilic indicator, tetramethylrhodamine methyl ester (TMRM; ThermoFisher Scientific, Waltham, MA) was used to determine mitochondrial membrane potential, at a concentration of 50 nM. The greater the potential, the more dye accumulates and the greater the signal intensity at any given pixel. Reactive oxygen species (ROS) generation in tubules was measured using dihydroethidium (Het; ThermoFisher Scientific), at a concentration of 5 µM. As HEt fluoresces on oxidization by superoxide, the fluorescence signal increases in proportion to the rate of ROS production. At 720 nm excitation, the autofluorescence signal emitted between 435 and 485 nm (cyan) arises predominantly from mitochondrial reduced nicotinamide adenine dinucleotide (NADH), enabling monitoring of change in mitochondrial redox status. Cell viability was assessed using Calcein AM (ThermoFisher Scientific). In live cells, Calcein AM is converted to a green-fluorescent calcein by intracellular esterases and detected as a green autofluorescence emission pattern at 800 nm excitation.

Changes in cell viability, mitochondrial membrane potential, ROS production and NADH redox state of proximal tubular cells were studied in slices incubated in either 1) physiologic saline solution (PSS); 2) sham serum; 3) septic serum; or 4) septic serum coincubated with the antioxidant, 4-hydroxy-2,2,6,6-tetramethyl-piperidin-1-oxyl (4-hydroxy-TEMPO) at a final concentration of 1 nM (Sigma, Gillingham, Dorset, United Kingdom). Serum was taken at 24 hours as this timepoint corresponded to significant differences in renal function. Serum was pooled from six rats and diluted to a 1:3 ratio in PSS. The septic serum was obtained from animals that underwent a similar experimental protocol of sepsis induction and fluid resuscitation, albeit with a different batch of slurry. The confocal experiments were performed within 2 years of the in vivo work. Confocal images were taken every 10 minutes for a total of 60 minutes. A total of 7–10 sets of images were taken to assess changes in TMRM, Het, and NADH, and three sets to assess changes in calcein for cell viability.

Mean fluorescent intensity was expressed as a percentage of mean fluorescent intensity at baseline. Images were taken at 10-minute intervals, focusing on different areas of the slice to avoid damage (bleaching) to the slice from repeated imaging of the same field.

### Uncoupling Protein Quantification by Western Blot

Protein was extracted and estimated from renal tissue or renal slices. Samples (20 µg protein) were electrophoresed at 100 V for 1 hour through a 12% or 15% sodium dodecyl sulfate polyacrylamide gel electrophoresis gel under reducing conditions. Proteins were transferred to a polyvinylidene difluoride membrane (GE Healthcare, Amersham, Buckinghamshire, United Kingdom) at 10 V for 45 minutes and then blocked for 1 hour in 5% milk/1% tris-buffered saline. This membrane was then incubated with goat anti-mouse immunoglobulin-G polyclonal uncoupling protein (UCP-2) antibody (Santa Cruz, Dallas, TX) at 1:250 in 5% bovine serum albumin (BSA; Sigma) in phosphate-buffered saline overnight at 4^o^C. Following incubation with the primary antibody, horseradish peroxidase rabbit anti-goat IgG (Sigma) was added at 1:3000 in 5% milk for 1 hour. Activity was detected using ECL Plus substrate (GE Healthcare). Four animals were randomly selected from each of the sham and sepsis groups at 6 and 24 hours for analysis.

To demonstrate evidence of renal oxidative stress in vivo, we measured levels of isoprostanes within the urine of septic animals and sham-operated animals at 24 hours. The commercially available OxiSelectTM 8- iso- Prostaglandin F2α Enzyme-Linked Immunosorbent Assay Kit (Cell Bio Labs, San Diego, CA) was used. Assays were run as per manufacturer’s protocol.

### Statistical Analyses

Analyses were performed and graphs drawn using Graphpad Prism Version 5.0d (GraphPad Software, La Jolla, CA). In view of relatively small sample sizes, nonparametric tests were used. Continuous variables are presented as median (interquartile range). Differences in continuous variables between groups were compared using Mann Whitney *U* test or Kruskal-Wallis test with post hoc Dunn test for more than two groups. For in vivo data, comparisons were made between sham-operated and septic animals at 6 and 24 hours. For ex vivo data, comparisons were made between slices incubated in PSS, sham serum, septic serum, or septic serum with 4-OH-TEMPO using Kruskal-Wallis tests with post hoc Dunn test. A *p* value less than 0.05 was considered statistically significant.

## RESULTS

### Physiology and Biochemistry of the In Vivo Peritonitis Model

For details, see **Supplementary Fig. 1** (Supplemental Digital Content 2, http://links.lww.com/CCM/D124--**legend**, Supplemental Digital Content 3, http://links.lww.com/CCM/D125) and **Table 1**.

**TABLE 1.**
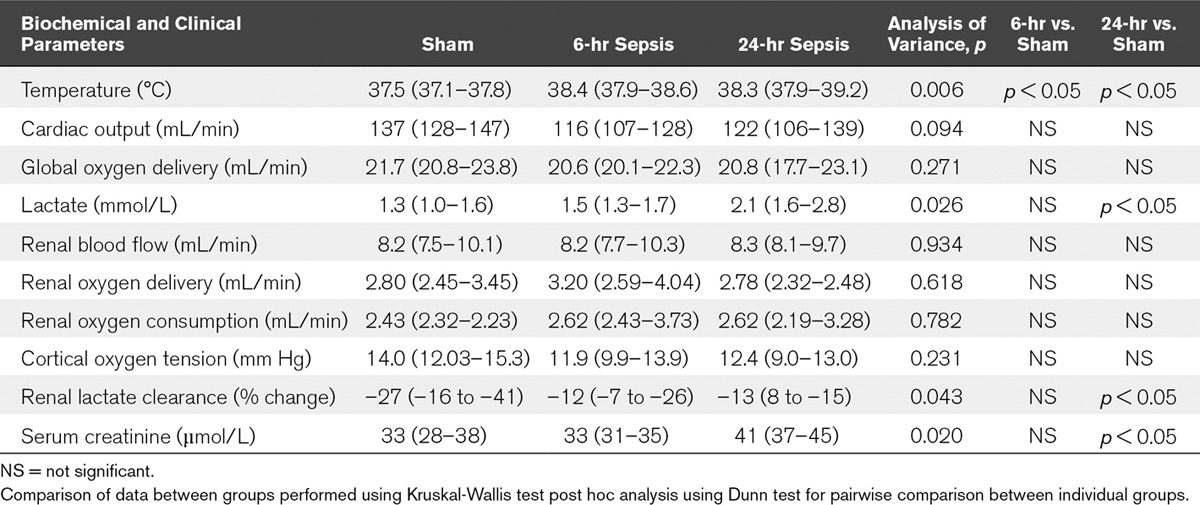
Physiologic and Biochemical Parameters After 6- and 24-Hour Sepsis

At baseline, all measured variables were similar. All six sham-operated animals survived to study end. All septic animals (*n* = 6–10 per group) survived the 6- and 24-hour experiments, but two animals were peri-mortem at 24 hours and thus excluded from analysis.

Compared with sham-operated animals whose body temperature was normothermic (37.5 [37.1–37.8]^o^C) throughout, the septic animals were febrile at both 6 hours (38.4 [37.9–38.6]^o^C) and 24 hours (38.3 [37.9–39.2]^o^C) (*p* = 0.006). Arterial lactate concentration was elevated only at 24 hours (2.1 [1.6–2.8] mmol/L) in septic animals compared with sham-operated animals (1.3 [1.0–1.6] mmol/L) (*p* = 0.026). There was no significant difference between sham-operated animals and septic animals (at either time point) in cardiac output or global oxygen delivery (Do_2_).

There was no statistically significant difference between sham-operated and septic animals at either timepoint in renal DO_2_, oxygen consumption (VO_2_), or renal cortical oxygen tension (PO_2_). At 24 hours serum creatinine (41 [37–45] µmol/L) was elevated compared with sham-operated animals (33 [28–38] µmol/L) (*p* = 0.020). Renal lactate clearance reduced in septic animals at 24 hours (–13 [8 to –15]%) compared with sham-operated animals (–27 [–16 to –41]%) (*p* = 0.043).

### Microscopy

For details, see **Supplementary Fig. 2**, Supplemental Digital Content 5, http://links.lww.com/CCM/D127--legend, Supplemental Digital Content 3, http://links.lww.com/CCM/D125).

Tubular injury on light microscopy was subtle and focal. There was some loss of the brush border microvilli and mild tubular dilation at both 6 and 24 hours. TUNEL stain revealed minimal cell death. Where present, cell death was localized to PTECs. Representative images from electron microscopy showed normal tubular epithelial mitochondrial structure in both sham-operated and septic animals at 24 hours.

### Multiphoton Confocal Imaging

For details, see **Supplementary Fig. 3** (Supplemental Digital Content 6, http://links.lww.com/CCM/D128--legend, Supplemental Digital Content 3, http://links.lww.com/CCM/D125) and **Table 2**.

**TABLE 2.**
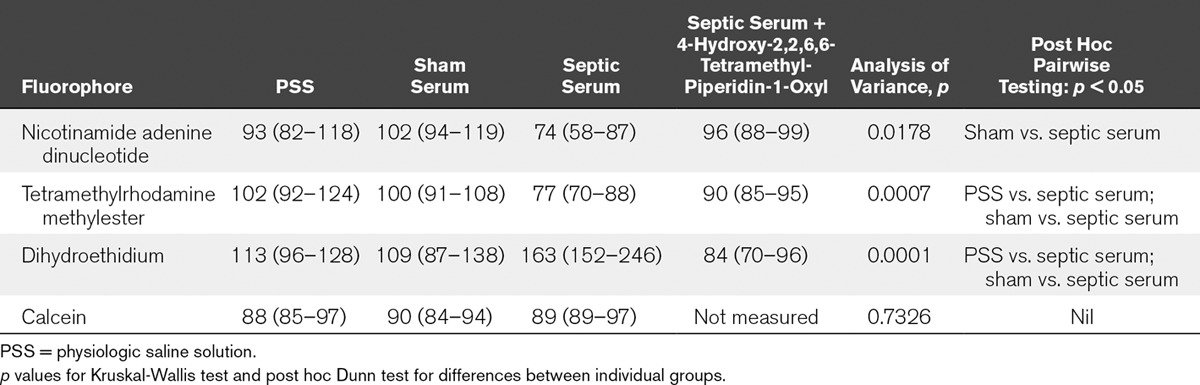
Percentage Change in Mean Fluorescence Intensity at 60 Minutes Compared With Baseline Following Incubation in Physiologic Saline Solution, Sham Serum, Septic Serum, or Septic Serum Coincubated With 4-Hydroxy-2,2,6,6-Tetramethyl-Piperidin-1-Oxyl

Using multiphoton confocal imaging of live kidney slices, cell viability, mitochondrial NADH redox state, membrane potential, and tubular ROS levels remained stable over the 60-minute time incubation period in both PSS and sham serum. However, incubation of live kidney slices in septic serum resulted in a significant progressive fall in NADH redox state in the mitochondria of PTECs compared with sham serum (74 [58–87]% vs. 102 [94–119]%, respectively; *p* = 0.018). A progressive fall in mitochondrial membrane potential was seen following incubation of live kidney slices in septic serum compared with sham serum (77 [70–88]% vs. 100 [91–108]%, respectively; *p* = 0.0007). Incubation of live kidney slices in septic serum and 4-OH-TEMPO was not associated with any changes in either PTEC NADH redox state (96 [88–99]%) or mitochondrial membrane potential (90 [85–95]%) compared with incubation of live kidney slices in sham serum (*p* > 0.05).

ROS levels were significantly increased on incubation of live kidney slices with septic serum (163 [152–256]%) compared with sham serum (109 [87–138]%) in PTECs; *p* = 0.0001). Incubation of live kidney slices in septic serum and 4-OH-TEMPO was not associated with any change in PTEC ROS levels (84 [70–96%]; *p* > 0.05). No change in calcein uptake was seen in any groups over the 60-minute period, suggesting no change in cell viability (*p* = 0.733).

### Renal UCP-2 Quantification

**Figure 2.**
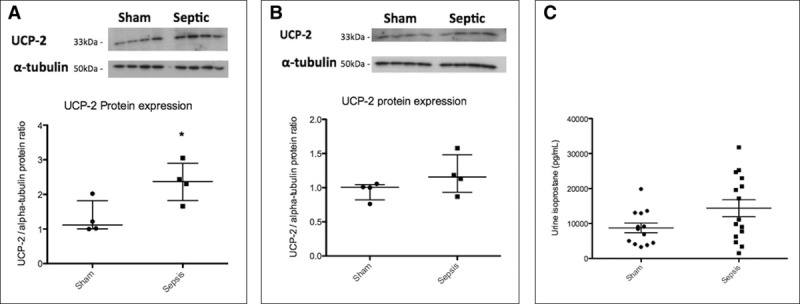
Renal uncoupling protein by Western blot and urine isoprostane levels. *Circles* and *squares* represent individual data points in the scatter plot. **A**, Uncoupling protein-2 (UCP-2) in kidney homogenate from sham-operated and 24-hour septic animal. The Western blot demonstrates a clear increase in renal UCP-2 expression in septic animals (1.56-fold increase) compared with sham-operated animals (*p* = 0.05). **B**, UCP-2 in kidney slice exposed to sham and septic serum. The Western blot demonstrates a modest increase in renal UCP-2 expression in live kidney slices exposed to septic serum (1.11-fold increase) compared with sham serum (*p* = 0.10). **C**, Urine isoprostane levels from sham-operated and septic animals at 24 hours. There is an increase in urine isoprostane levels in septic animals compared with sham-operated animals at 24 hours, which approaches statistical significance (*p* = 0.06).

Western blot revealed a 1.56-fold increase in UCP-2 concentration in kidneys taken from septic rats (*p* = 0.05 compared with sham-operated) (**Fig. 2*A*** and ***B***). UCP-2 levels rose modestly (1.11-fold increase) in live kidney slices exposed to septic serum over 60 minutes compared with sham serum, though this did not reach statistical significance (*p* = 0.10).

We measured urine isoprostane levels in sham-operated and septic animals at 24 hours (**Fig. 2*C***). Two of the samples run were spuriously high, which we excluded and analyzed the results. The levels of urine isoprostane levels are higher in 24-hour septic animals compared with sham-operated animals, though does not reach statistical significance (11,125 [6,254–31,769] pg/mL vs. 8,409 [4,285–13,014] pg/mL; *p* = 0.06).

## DISCUSSION

Our in vivo model of resuscitated fecal peritonitis recapitulates many of the features of human sepsis, with an early rise in core temperature followed by a rise in arterial lactate and organ dysfunction. Consistent with published data, our model confirms that sepsis-induced AKI can still occur notwithstanding the absence of structural tubular injury, decreases in RBF and oxygen delivery, and hemodynamic instability (2–4). After 24 hours of sepsis, renal VO_2_, DO_2_, and cortical oxygenation were all stable. Renal mitochondrial ultrastructure was also normal, again suggestive of a functional rather than structural pathophysiologic mechanism.

Renal oxygenation depends on the balance between local DO_2_ and VO_2_. In health, renal VO_2_ depends on renal DO_2_, with oxygen extraction remaining stable over a wide range of local blood flow (17). Tubular reabsorption of filtered sodium is the major determinant of renal VO_2_ (18), while tubular transport processes are dependent on the filtered load (19, 20). Any decrease in renal function (i.e., glomerular filtration rate [GFR], as reflected in the rise in serum creatinine at 24 hr) would decrease filtered sodium and thus reduce tubular VO_2_. Despite this, renal VO_2_ remained stable at 24 hours. In conjunction with the reduction in renal function at 24 hours (rise in creatinine, reduction in lactate clearance), this suggests that renal oxygen consumption may be partially redirected away from adenosine triphosphate (ATP) production (i.e., oxidative phosphorylation). The rise in UCP-2 protein also supports the possibility of increased uncoupling of mitochondrial respiration.

The investigations on renal proximal tubular cell mitochondrial function within the live kidney slices incubated with septic serum supports these in vivo findings. Despite cell viability being maintained, the falls in tubular NADH and mitochondrial membrane potential are also consistent with increased uncoupling of mitochondrial oxygen utilization away from ATP production. Coincubation with the ROS scavenger TEMPO attenuated these changes and prevented the rise in ROS levels in the PTECs, implicating excessive oxidant stress as an important pathologic mechanism. During sepsis, high levels of ROS and reactive nitrogen species are produced, and these may overwhelm antioxidant capacity with resultant inhibition of, and damage to, the electron transport chain (21, 22). Mitochondria exposed to nonmitochondrial ROS become a source of ROS themselves (23). This positive feedback loop of ROS-induced-ROS is likely to culminate in uncoupled respiration, dysregulation, and damage to mitochondria. The reduction in membrane potential may however be a useful negative feedback mechanism to limit the amount of harmful ROS produced (24). However, excessive ATP depletion caused by the uncoupling of oxidative phosphorylation is associated with collapse of the electrochemical gradient across the mitochondrial membrane and release of ROS (25). While nonsignificant increase in renal UCP-2 protein was seen in the short time period (60 min) during which the live kidney slices were incubated in septic serum, there may be an increase in activity.

To demonstrate evidence of increased renal oxidative stress in vivo, measurement of urine F2-isoprostanes in 24-hour sham-operated and septic animals was performed. Measurement of F2-isoprostanes (a product of free radical-catalyzed peroxidation of arachidonic acid) has emerged as one of the most reliable approaches to assess oxidative stress status in vivo (26). The levels of urine isoprostane levels are higher in 24-hour septic animals compared with sham-operated animals (**Fig 2*C***), though does not reach statistical significance (*p* = 0.06).

The association between increased ROS, mitochondrial dysfunction, and renal dysfunction in sepsis in vivo has been described by others (27, 28). We have unified the in vivo and in vitro experiments to arrive at the same conclusion—therefore adding strength to the hypothesis. We have demonstrated that the changes seen both in vivo and in vitro are flow-independent. Furthermore, the changes induced in the in vitro live kidney slice experiments are from septic serum obtained from the in vivo experiments.

The change in mitochondrial function seen on incubation of live kidney slices in serum from septic rats suggests a circulating mediator(s) is responsible for changes seen in sepsis. Others have also demonstrated that renal proximal tubular cells exposed to septic serum are prone to mitochondrial dysfunction (decrease in mitochondrial membrane potential, ATP depletion, and increased superoxide and peroxynitrite levels), and cytotoxicity (29). Other cell types including endothelial cells (30), cardiac myocytes (31), and fibroblasts (32) are also susceptible to mitochondrial dysfunction on exposure to septic serum. The precise mediators within septic serum that are responsible for these changes are as yet unidentified.

Previous work from our laboratory in this rat model has demonstrated a temporal change in markers of renal injury (urine NGAL, KIM-1, calbindin), followed by a rise in a marker of cell cycle arrest (urine IGFBP7) and, finally, by markers of decreased filtration (serum creatinine and cystatin C) (13). We hypothesized a model where there is direct renal tubular injury from circulating inflammatory mediators, followed by cell cycle arrest as a means of reducing tubular cell replication and, then by a reduction in function. Significant reductions in ATP result in cell death via several mechanisms, including opening of the mitochondrial transition pore (33). As such, reduction in cell cycling, a highly energy-dependent process, would facilitate cell survival in conditions where oxidative phosphorylation is reduced. Furthermore, the “functional shutdown” of GFR and tubular function may initiate various protective mechanisms including conservation of circulating volume, though at the expense of functionality (34). Mitochondrial dysfunction/uncoupling may represent an early manifestation of renal injury in vivo, which is demonstrated by the rapid changes in mitochondrial functionality seen in our healthy kidney slices incubated with septic serum.

Although the confocal work focuses on the proximal tubules, we acknowledge that the etiology of kidney dysfunction is likely to be multifactorial, and other renal cell types may be also involved. However, recent work strongly points to a key role of the PTEC as a site of injury. Markers of proximal tubule injury were elevated (e.g., IGFBP-7), whereas changes to distal tubular injury biomarkers were not as pronounced (13, 35).

We avoided antibiotic use to avoid potential direct renal toxicity (36). Serum creatinine in the septic animals rose by 50% and did not formally measure creatinine clearance. Although the rise in creatinine is less than that seen clinically, the requirement for significant fluid resuscitation may have a dilution effect (37). Furthermore, creatinine production is reduced during early sepsis in rodents (38). Of note, in this model, animals with a greater than 50% rise in serum creatinine at 24 hours tend not to survive.

The time course of ROS generation in slices exposed to septic serum is significantly shorter compared with upregulation of UCP-2 protein, which was not upregulated until 24 hours in vivo. The in vitro experiments, however, are a proof of concept study to demonstrate that renal PTECs are capable of producing ROS and undergo changes consistent with uncoupling that are independent of oxygen delivery. These experiments cannot be directly compared with the in vivo experiments due to different conditions inherent to the nature of the experiments. Our in vitro mitochondrial studies were performed at levels of oxygen (room air) that are “nonphysiological.” Although clear differences were seen between kidney slices incubated with either septic or sham serum, it is yet to be determined whether the same holds true at more physiologic levels of oxygenation. Furthermore, the renal PTECs are in direct contact with serum in the in vitro experiment.

We are not able to comment specifically on mitochondrial mass or mitophagy, as these were not measured. However, it is unlikely that this would occur so rapidly as to cause a significant decrease in mitochondrial mass in 1 hour. Although we did not assess in vivo ATP turnover, we propose that mitochondrial uncoupling would divert oxygen consumption away from ATP production. The age of rats we use are equivalent to young adults in humans. We acknowledge that the age of rats may influence immune and bioenergetic function of mitochondria. However, younger rats are used more commonly in experimental medicine due to cost implications, which is a limitation inherent to many published animal studies.

Despite these limitations, our study has a number of strengths and novel findings. We demonstrate many findings consistent with the reported literature, including lack of change in renal hemodynamics and histology, notwithstanding a rise in serum creatinine and decreased renal lactate utilization. Septic rats were pyrexic, and renal UCP-2 protein was increased. As uncoupled respiration leads to increased heat production, this may be an important mechanism underlying the generation of fever in sepsis. Of note, circulating mediators within septic serum could induce changes consistent with mitochondrial uncoupling within PTECs that are mediated via excess ROS production. Using complimentary in vivo and in vitro studies, we describe a mechanism that may potentially explain the paradigm of sepsis-induced AKI and offer new therapeutic approaches.

## Supplementary Material

## References

[1] Schrier RW Wang W Acute renal failure and sepsis. N Engl J Med 2004; 351:159 169 15247356 10.1056/NEJMra032401

[2] Langenberg C Bagshaw SM May CN The histopathology of septic acute kidney injury: A systematic review. Crit Care 2008; 12:R38 18325092 10.1186/cc6823 PMC2447560

[3] Lerolle N Nochy D Guérot E Histopathology of septic shock induced acute kidney injury: Apoptosis and leukocytic infiltration. Intensive Care Med 2010; 36:471 478 19924395 10.1007/s00134-009-1723-x

[4] Takasu O Gaut JP Watanabe E Mechanisms of cardiac and renal dysfunction in patients dying of sepsis. Am J Respir Crit Care Med 2013; 187:509 517 23348975 10.1164/rccm.201211-1983OC PMC3733408

[5] Brenner M Schaer GL Mallory DL Detection of renal blood flow abnormalities in septic and critically ill patients using a newly designed indwelling thermodilution renal vein catheter. Chest 1990; 98:170 179 2361386 10.1378/chest.98.1.170

[6] Lucas CE Rector FE Werner M Altered renal homeostasis with acute sepsis. Clinical significance. Arch Surg 1973; 106:444 449 4696717 10.1001/archsurg.1973.01350160062010

[7] Rector F Goyal S Rosenberg IK Sepsis: A mechanism for vasodilatation in the kidney. Ann Surg 1973; 178:222 226 4723431 10.1097/00000658-197308000-00021 PMC1355637

[8] Wan L Langenberg C Bellomo R Angiotensin II in experimental hyperdynamic sepsis. Crit Care 2009; 13:R190 19948019 10.1186/cc8185 PMC2811902

[9] Prowle JR Molan MP Hornsey E Measurement of renal blood flow by phase-contrast magnetic resonance imaging during septic acute kidney injury: A pilot investigation. Crit Care Med 2012; 40:1768 1776 22487999 10.1097/CCM.0b013e318246bd85

[10] Murugan R Karajala-Subramanyam V Lee M; Genetic and Inflammatory Markers of Sepsis (GenIMS) Investigators: Acute kidney injury in non-severe pneumonia is associated with an increased immune response and lower survival. Kidney Int 2010; 77:527 535 20032961 10.1038/ki.2009.502 PMC2871010

[11] Carré JE Orban JC Re L Survival in critical illness is associated with early activation of mitochondrial biogenesis. Am J Respir Crit Care Med 2010; 182:745 751 20538956 10.1164/rccm.201003-0326OC PMC2949402

[12] Dyson A Rudiger A Singer M Temporal changes in tissue cardiorespiratory function during faecal peritonitis. Intensive Care Med 2011; 37:1192 1200 21533572 10.1007/s00134-011-2227-z

[13] Arulkumaran N Sixma ML Jentho E Sequential analysis of a panel of biomarkers and pathologic findings in a resuscitated rat model of sepsis and recovery. Crit Care Med 2017; 45:e821 e830 28430696 10.1097/CCM.0000000000002381 PMC5511729

[14] Rudiger A Dyson A Felsmann K Early functional and transcriptomic changes in the myocardium predict outcome in a long-term rat model of sepsis. Clin Sci (Lond) 2013; 124:391 401 22988837 10.1042/CS20120334

[15] Whitehouse T Stotz M Taylor V Tissue oxygen and hemodynamics in renal medulla, cortex, and corticomedullary junction during hemorrhage-reperfusion. Am J Physiol Renal Physiol 2006; 291:F647 F653 16525156 10.1152/ajprenal.00475.2005

[16] Hall AM Unwin RJ Parker N Multiphoton imaging reveals differences in mitochondrial function between nephron segments. J Am Soc Nephrol 2009; 20:1293 1302 19470684 10.1681/ASN.2008070759 PMC2689904

[17] Levy MN Effect of variations of blood flow on renal oxygen extraction. Am J Physiol 1960; 199:13 18 14416431 10.1152/ajplegacy.1960.199.1.13

[18] Kiil F Aukland K Refsum HE Renal sodium transport and oxygen consumption. Am J Physiol 1961; 201:511 516 13755902 10.1152/ajplegacy.1961.201.3.511

[19] Torelli G Milla E Faelli A Energy requirement for sodium reabsorption in the *in vivo* rabbit kidney. Am J Physiol 1966; 211:576 580 5927884 10.1152/ajplegacy.1966.211.3.576

[20] Swärd K Valsson F Sellgren J Differential effects of human atrial natriuretic peptide and furosemide on glomerular filtration rate and renal oxygen consumption in humans. Intensive Care Med 2005; 31:79 85 15565364 10.1007/s00134-004-2490-3

[21] Boczkowski J Lisdero CL Lanone S Endogenous peroxynitrite mediates mitochondrial dysfunction in rat diaphragm during endotoxemia. FASEB J 1999; 13:1637 1646 10463956 10.1096/fasebj.13.12.1637

[22] Alvarez S Boveris A Mitochondrial nitric oxide metabolism in rat muscle during endotoxemia. Free Radic Biol Med 2004; 37:1472 1478 15454287 10.1016/j.freeradbiomed.2004.06.034

[23] Quoilin C Mouithys-Mickalad A Lécart S Evidence of oxidative stress and mitochondrial respiratory chain dysfunction in an *in vitro* model of sepsis-induced kidney injury. Biochim Biophys Acta 2014; 1837:1790 1800 25019585 10.1016/j.bbabio.2014.07.005

[24] Echtay KS Roussel D St-Pierre J Superoxide activates mitochondrial uncoupling proteins. Nature 2002; 415:96 99 11780125 10.1038/415096a

[25] Reid AB Kurten RC McCullough SS Mechanisms of acetaminophen-induced hepatotoxicity: Role of oxidative stress and mitochondrial permeability transition in freshly isolated mouse hepatocytes. J Pharmacol Exp Ther 2005; 312:509 516 15466245 10.1124/jpet.104.075945

[26] Montuschi P Barnes PJ Roberts LJ 2nd Isoprostanes: Markers and mediators of oxidative stress. FASEB J 2004; 18:1791 1800 15576482 10.1096/fj.04-2330rev

[27] Holthoff JH Wang Z Seely KA Resveratrol improves renal microcirculation, protects the tubular epithelium, and prolongs survival in a mouse model of sepsis-induced acute kidney injury. Kidney Int 2012; 81:370 378 21975863 10.1038/ki.2011.347 PMC3326404

[28] Patil NK Parajuli N MacMillan-Crow LA Inactivation of renal mitochondrial respiratory complexes and manganese superoxide dismutase during sepsis: Mitochondria-targeted antioxidant mitigates injury. Am J Physiol Renal Physiol 2014; 306:F734 F743 24500690 10.1152/ajprenal.00643.2013 PMC3962604

[29] Pathak E MacMillan-Crow LA Mayeux PR Role of mitochondrial oxidants in an *in vitro* model of sepsis-induced renal injury. J Pharmacol Exp Ther 2012; 340:192 201 22011433 10.1124/jpet.111.183756 PMC3251020

[30] Boulos M Astiz ME Barua RS Impaired mitochondrial function induced by serum from septic shock patients is attenuated by inhibition of nitric oxide synthase and poly(ADP-ribose) synthase. Crit Care Med 2003; 31:353 358 12576936 10.1097/01.CCM.0000050074.82486.B2

[31] Martin L Peters C Schmitz S Soluble heparan sulfate in serum of septic shock patients induces mitochondrial dysfunction in murine cardiomyocytes. Shock 2015; 44:569 577 26529654 10.1097/SHK.0000000000000462

[32] Trentadue R Raffaella T Fiore F Induction of mitochondrial dysfunction and oxidative stress in human fibroblast cultures exposed to serum from septic patients. Life Sci 2012; 91:237 243 22820545 10.1016/j.lfs.2012.06.041

[33] Kantrow SP Tatro LG Piantadosi CA Oxidative stress and adenine nucleotide control of mitochondrial permeability transition. Free Radic Biol Med 2000; 28:251 260 11281292 10.1016/s0891-5849(99)00238-5

[34] Thurau K Boylan JW Acute renal success. The unexpected logic of oliguria in acute renal failure. Am J Med 1976; 61:308 315 961698 10.1016/0002-9343(76)90365-x

[35] Emlet DR Pastor-Soler N Marciszyn A Insulin-like growth factor binding protein 7 and tissue inhibitor of metalloproteinases-2: Differential expression and secretion in human kidney tubule cells. Am J Physiol Renal Physiol 2017; 312:F284 F296 28003188 10.1152/ajprenal.00271.2016 PMC5336590

[36] Peng ZY Wang HZ Srisawat N Bactericidal antibiotics temporarily increase inflammation and worsen acute kidney injury in experimental sepsis. Crit Care Med 2012; 40:538 543 21926582 10.1097/CCM.0b013e31822f0d2e PMC3254710

[37] Prowle JR Leitch A Kirwan CJ Positive fluid balance and AKI diagnosis: Assessing the extent and duration of ‘creatinine dilution’. Intensive Care Med 2015; 41:160 161 25398305 10.1007/s00134-014-3538-7

[38] Doi K Yuen PS Eisner C Reduced production of creatinine limits its use as marker of kidney injury in sepsis. J Am Soc Nephrol 2009; 20:1217 1221 19389851 10.1681/ASN.2008060617 PMC2689892

