# Supplementary material for: Renal Tubular Cell Mitochondrial Dysfunction Occurs Despite Preserved Renal Oxygen Delivery in Experimental Septic Acute Kidney Injury
Source: Crit Care Med. 2018 Mar 14;46(4):e318–25. doi: 10.1097/CCM.0000000000002937 (PMC5856355; doi:10.1097/CCM.0000000000002937)
Supplement: Supplementary file 1 [file ccm-46-e318-s001.doc]

**Supplementary data**

**Methods**

All invasive and imaging techniques were performed under brief general anesthesia and postoperative analgesia. Following tunneled internal jugular central venous catheter placement, rats were placed in individual cages mounted on a tether/swivel system to secure the intravenous catheter, enable fluid resuscitation, and allow unimpeded movement with free access to food and water. Twenty-four hours later, sepsis was induced by intraperitoneal injection of fecal slurry (Figure 1). A similar procedure was avoided in sham animals to prevent inadvertent bowel perforation.

The fluid administered following the onset of sepsis was 10ml/kg/h based on previous experiments within our lab . As the severity of this model was greater than the current experiment, it remains very unlikely that in the current experiment we have ‘under-dosed’ intravenous fluids. The rate of fluid administration was increased to 20ml/kg/h during the period of the laparotomy (typically less than an hour) to compensate for increased insensible losses.

**Fecal peritonitis**

Previous studies from our group used fecal slurry which was obtained from rats and the model is essentially similar albeit with some modifications made over time (e.g. introduction of echocardiography and differences in the type/dose of fecal slurry used) .

We moved from rat slurry to a human fecal slurry preparation in approximately 2012 with two major advantages; (i) ready availability of sample (only simple defrosting needed) and homogeneity (hundreds of samples can be produced from a single stock pooled from three donors). Bigger doses of the same batch will increase the illness severity and mortality, so the dose selected is used to reflect the severity needs of the mode. For the current study, we aimed for a survival model so a dose of 3 ml/kg was used, resulting in a <10% mortality at 24 hrs. By contrast, our recent manuscript, used a dose of 4 ml/kg, which generated a 20% mortality. Method of preparation of human fecal slurry is detailed in the supplementary data of our related manuscript .

Different batches of human fecal slurry were used for the in vivo and ex vivo experiments, whilst maintaining a similar experimental protocol of sepsis induction and fluid resuscitation. Batch to batch variation in fecal slurry may result in random variability but not systematic bias, which is an inherent limitation associated with using stool from a healthy donor. These are inherent limitations associated with using stool from a healthy donor (whether that donor is a human or a rat), that could introduce random variability but not systematic bias.

**Renal cortical oxygen tension**

After laparotomy, a 22-gauge needle was used to puncture the renal capsule at the mid-pole. A fiberoptic optode (250μm diameter) connected to an Oxylite monitoring system (Oxford Optronix, Didcot, Oxon, UK) was inserted to a depth of 4-5 mm and subsequently withdrawn to a depth of 1-2 mm to prevent anomalous measurements due to any local hematoma. This enabled continuous tissue oxygen tension (tPO_2_) monitoring within the renal cortex (Figure 1). Measurements were recorded onto a computer using a 16-channel Powerlab system and Chart 5 acquisition software (AD Instruments, Chalgrove, Oxon, UK).

These oxygen sensors are pre-calibrated within the range 0-200 mmHg (0-26.7 kPa). Short excitatory pulses of light (475 nm) are emitted along the fiberoptic cable to a platinum-complex fluorophore situated at the sensor tip. Upon interaction with oxygen, the fluorophore emits light (600 nm) recorded at the detection unit. The lifetime of the returned light is inversely proportional to local PO_2_, according to the Stern-Volmer equation:

τ_0_/ τ_1_ = 1 + (κ_q_ x τ_0_ x {O_2_})

where τ_0_ is the decay time at zero oxygen, τ_1_ is the decay time at a specific oxygen concentration (O_2_) and κ_q_ is a quenching rate constant that denotes the probability of a photoluminescent molecule and oxygen molecule colliding. As luminescence decay is longer at a lower PO_2_, accurate measurements can be made within the physiological range (0-8 mmHg (0-1.1 kPa)).

**Renal blood flow measurement**

The left renal artery was isolated by careful blunt dissection. An ultrasonic flow probe (Transonic Systems, Ithaca, NY, USA) of 1 mm diameter was coated in a water-soluble lubricant and placed around the left renal artery to measure renal blood flow (RBF). Renal oxygen delivery (DO_2_ renal) was calculated using the following formula:

DO_2_ renal = 1.34 x RBF (L/min) x Hb (g/l) x SaO_2_

**Renal lactate clearance**

The left renal vein was isolated from surrounding tissue by careful blunt dissection. One ligature was placed around the renal vein, and another around the renal artery and vein. The renal vein was kinked using the ligature, punctured with an 18-gauge needle, and approximately 0.3 ml blood aspirated. The renal vein and artery were then ligated to prevent blood loss. A simultaneous blood sample was taken from the arterial line. Both were analyzed in a blood gas analyser (ABL-70, Radiometer, Copenhagen, Denmark).

Renal lactate clearance was calculated as:

(Renal vein - arterial lactate) x 100

Arterial lactate

***Ex vivo* assessment of mitochondrial function using confocal microscopy**

Using dyes or natural fluorophores, confocal microscopy allows detailed imaging of cellular physiologic processes in intact renal tissue sections. Multiphoton imaging uses a long wavelength excitation laser that permits greater tissue penetration compared to conventional single-laser confocal fluorescence microscopy. This technique can image live kidney slices in real-time in response to various insults or drugs. We previously reported the use of multiphoton imaging of freshly prepared rat kidney slices to investigate mitochondrial function in cells along the nephron in response to toxic stimuli, including chemical anoxia .

The left kidney was removed from an anesthetized healthy male Wistar rat and immediately placed in oxygenated ice-cold HEPES-buffered solution (118mM NaCl, 10mM NaHCO_3_, 4.7mM KCl, 1.44mM MgSO_4_, 1.2mM KH_2_ PO_4_ , 1.8mM CaCl_2_, 10mM HEPES, 5mM glucose, 5 mM sodium butyrate, 5mM pyruvate). The kidney was sliced in half along the transverse plane and mounted on a stage. Slices were cut at 200 μm in oxygenated ice-cold HEPES-buffered solution using a EMS 5000 Oscillating Tissue Slicer (Electron Microscopy Sciences, PA, USA).

Slices were then incubated in oxygenated HEPES-buffered solution at room temperature. They were placed singly in an open bath chamber (Harvard Apparatus, Edenbridge, Kent, UK) and secured with a slice anchor. Dyes and reagents were loaded using an on-stage perfusion system, and changes in signal imaged in real-time. Kidney sections were studied up to 6 hours following resection. Structures were identified by their characteristic morphology and location.

Slices were imaged using a Zeiss LSM 510 NLO axiovert microscope (Carl Zeiss, Welwyn Garden City, Herts, UK) coupled to a tunable Chameleon laser (Coherent Incorporated, Santa Clara, USA). The optimal excitation wavelength was based on two-photon excitation spectra . An internal detector captured emitted light. Image processing and analysis was performed using Zeiss LSM software and Image J software (National Institutes of Health, Bethesda, MD, http://rsb.info.nih.gov/ij/). To quantify fluorescence signals, regions of interest were drawn around tubules in a minimum of three different fields (imaged using a x40 objective). All values are expressed as mean fluorescence intensity per image pixel (arbitrary units, AU), within the relevant region of interest.

The cationic lipophilic indicator, tetramethyl rhodamine methyl ester (TMRM; (ThermoFisher Scientific, Waltham, MA) was used to determine mitochondrial membrane potential, at a concentration of 50 nM. The greater the potential, the more dye accumulates and the greater the signal intensity at any given pixel. ROS generation in tubules was measured using dihydroethidium (Het; ThermoFisher), at a concentration of 5 μM. As HEt fluoresces on oxidization by superoxide, the fluorescence signal increases in proportion to the rate of ROS production. At 720 nm excitation, the autofluorescence signal emitted between 435-485 nm (cyan) arises predominantly from mitochondrial NADH, enabling monitoring of change in mitochondrial redox status. The obtained image consists of striations of signal at the basal pole of the cell, matching the known distribution of mitochondria in the tubule and the signal seen with TMRM. Cell viability was assessed using Calcein AM (ThermoFisher). In live cells calcein AM is converted to a green-fluorescent calcein by intracellular esterases and detected as a green autofluorescence emission pattern at 800nm excitation.

Changes in cell viability, mitochondrial membrane potential, reactive oxygen species (ROS) production and the NADH redox state of proximal tubular cells were studied in slices incubated in either (i) physiological saline solution (PSS), (ii) sham serum, (iii) septic serum, or (iv) septic serum co-incubated with the antioxidant, TEMPO at 1nM (Sigma, Gillingham, Dorset, UK). Serum was taken at 24h as this timepoint corresponded to significant differences in renal VO_2_. Serum was pooled from x rats and diluted to a 1:3 ratio in PSS. Confocal images were taken every 10 min for a total of 60 min. A total of 7-10 sets of images were taken to assess changes in TMRM, Het, and NADH and 3 sets to assess changes in calcein for cell viability.

Mean fluorescent intensity was expressed as a percentage of mean fluorescent intensity at baseline. Images were taken at 10-min intervals, focusing on different areas of the slice to avoid damage (bleaching) to the slice from repeated imaging of the same field.

**References**

1.Rudiger A, Dyson A, Felsmann K*, et al.*: Early functional and transcriptomic changes in the myocardium predict outcome in a long-term rat model of sepsis: *Clin Sci (Lond)* 2013; 124:391-401

2.Brealey D, Karyampudi S, Jacques TS*, et al.*: Mitochondrial dysfunction in a long-term rodent model of sepsis and organ failure: *Am J Physiol Regul Integr Comp Physiol* 2004; 286:R491-7

3.Dyson A, Rudiger A, Singer M: Temporal changes in tissue cardiorespiratory function during faecal peritonitis: *Intensive Care Med* 2011; 37:1192-200

4.Arulkumaran N, Sixma ML, Jentho E*, et al.*: Sequential Analysis of a Panel of Biomarkers and Pathologic Findings in a Resuscitated Rat Model of Sepsis and Recovery: *Crit Care Med* 2017; 45:e821-e30

5.Whitehouse T, Stotz M, Taylor V*, et al.*: Tissue oxygen and hemodynamics in renal medulla, cortex, and corticomedullary junction during hemorrhage-reperfusion: *Am J Physiol Renal Physiol* 2006; 291:F647-53

6.Hall AM, Unwin RJ, Parker N*, et al.*: Multiphoton imaging reveals differences in mitochondrial function between nephron segments: *J Am Soc Nephrol* 2009; 20:1293-302
