# Supplementary figures and images for: Renal Tubular Cell Mitochondrial Dysfunction Occurs Despite Preserved Renal Oxygen Delivery in Experimental Septic Acute Kidney Injury
Source: Crit Care Med. 2018 Mar 14;46(4):e318–25. doi: 10.1097/CCM.0000000000002937 (PMC5856355; doi:10.1097/CCM.0000000000002937)

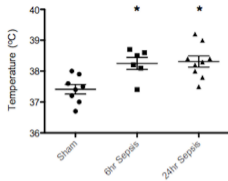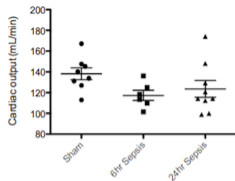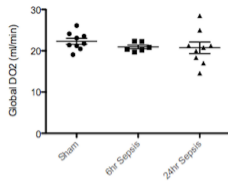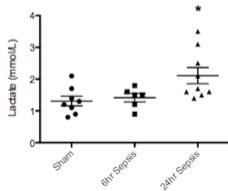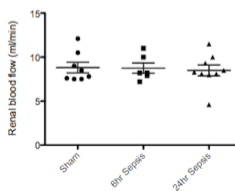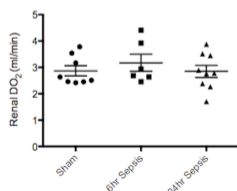

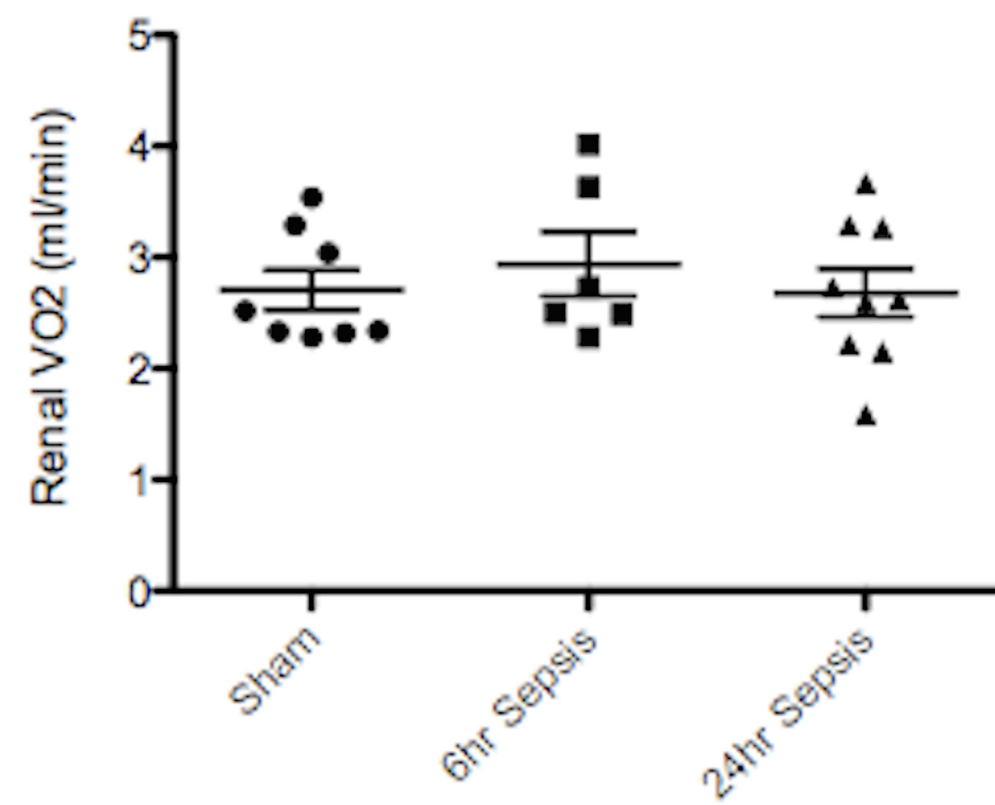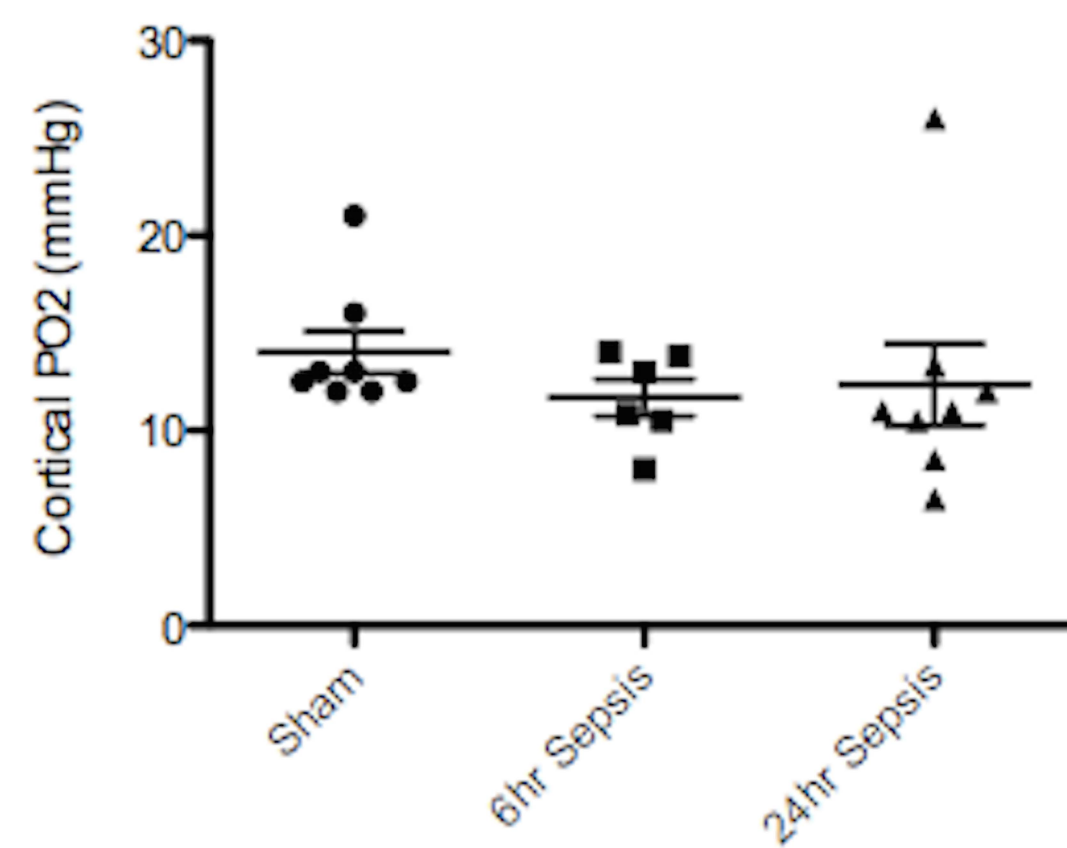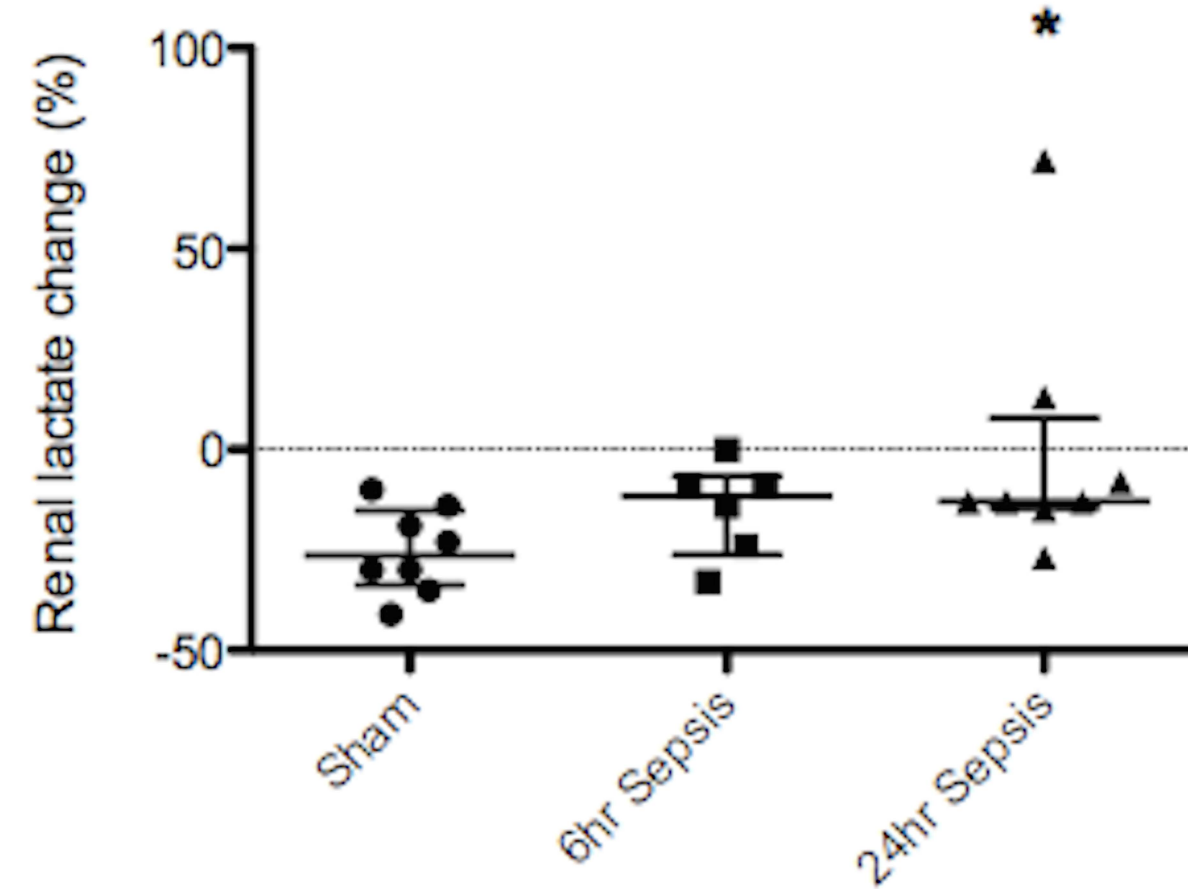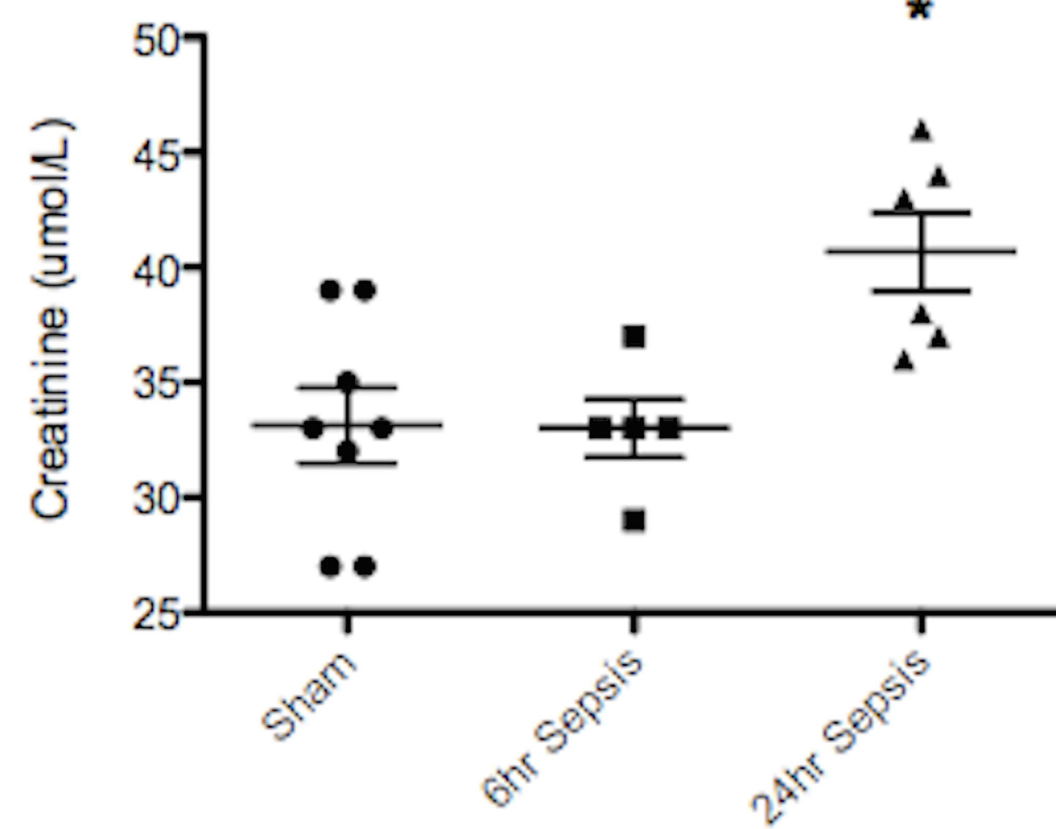

Supplement: Supplementary file 2 [file ccm-46-e318-s002.pdf]

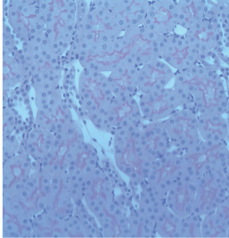

a.

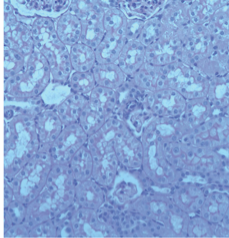

b.

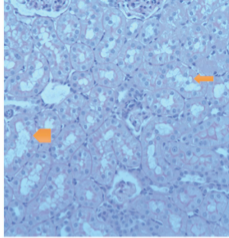

c.

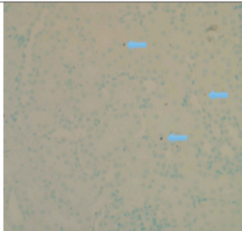

d.

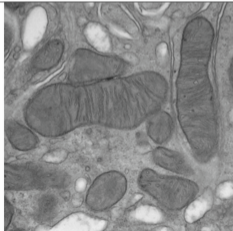

182-14 Rat kidney 322 sham\_027  
Tubule 11

e.

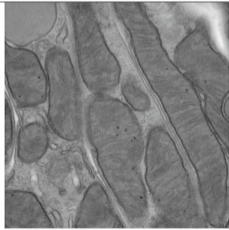

182-14 Rat kidney 321 Septic\_026  
Tubule 14

f.

Supplement: Supplementary file 4 [file ccm-46-e318-s004.pdf]

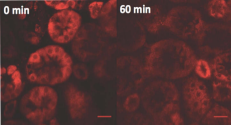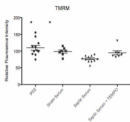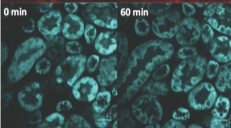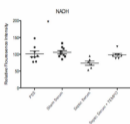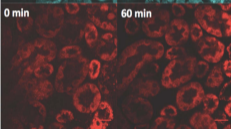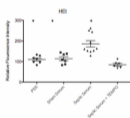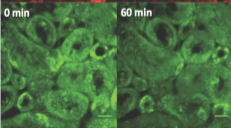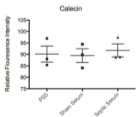

Supplement: Supplementary file 5 [file ccm-46-e318-s005.pdf]
