# Supplementary material for: Renal Tubular Cell Mitochondrial Dysfunction Occurs Despite Preserved Renal Oxygen Delivery in Experimental Septic Acute Kidney Injury
Source: Crit Care Med. 2018 Mar 14;46(4):e318–25. doi: 10.1097/CCM.0000000000002937 (PMC5856355; doi:10.1097/CCM.0000000000002937)
Supplement: Supplementary file 3 [file ccm-46-e318-s003.doc]

**Supplementary Figures**

**Figure 1: Physiological and biochemical data.**

Compared to sham-operated animals, septic animals show (a) rise in temperature at 6h and 24h (p=0.006), (b) non-significant change in cardiac output (p=0.094), (c) maintained global oxygen delivery (p=0.271) and (d) rise in serum lactate at 24h (p=0.026). Renal blood flow (e), renal oxygen delivery (f), renal oxygen consumption (g), and renal cortical oxygenation (h) were maintained. However, at 24h, renal clearance of lactate was reduced (p=0.043) and serum creatinine was elevated (p=0.02).

Comparison of data between groups performed using Kruskal-Wallis test with post-hoc Dunn’s test for pairwise comparison between individual groups.

* indicates p<0.05 compared to sham operated animals.

**Figure 2: Renal microscopy**

Light microscopy revealing (a) healthy proximal tubules from a sham-operated animal (x20 magnification); (b) mild injury of proximal tubules and loss of brush border after 6h of sepsis; and (c) mild injury of proximal tubules after 24h of sepsis. Predominant changes include loss of brush border, vacuolation of tubular cells (arrowhead), and mild tubular dilation (arrow)

(d) TUNEL stain of renal tissue taken after 24h sepsis shows occasional apoptotic cells (blue arrow). Apoptotic bodies seen mainly in proximal tubular epithelial cells. (x20 magnification).

(e-f) Electron microscopy revealing normal ultrastructure of mitochondria with preserved cristae in both sham-operated and septic animals (x40000 magnification) (scale bar=500nm)

**Figure 3: Changes in confocal fluorescence signal in live kidney slices at** **90 min post-addition of septic serum**

(a) Loading with the mitochondrial membrane potential–dependent dye tetramethyl rhodamine methyl ester (TMRM) and excitation at 860 nm. Mitochondrial membrane potential (MMP) in PTECs fell on with septic serum (p<0.05 compared to sham serum). The fall in MMP was abrogated by co-incubation with the antioxidant 4-OH-TEMPO.

(b) Mitochondrial NADH visualized in proximal tubular epithelial cells at 720 nm excitation. Incubation with septic serum resulted in a significant drop in reduced NADH (p<0.05 compared to sham serum). The fall in NADH redox state was abrogated by co-incubation with the antioxidant 4-OH-TEMPO.

(c) Loading with the reactive oxygen species (ROS)-sensitive dye, dihydroethidium (HEt) and excitation at 720nm. Increased ROS production is seen in proximal tubular epithelial cells over 90 min following exposure to septic serum (p<0.05 compared to sham serum). This was prevented by co-incubation with 4-OH-TEMPO

(d) Loading with calcein and excitation at 800 nm to demonstrate cell viability. No change in calcein uptake is seen over a 90 minute period after incubation with septic serum, suggesting no change in cell viability (p=0.733).

* p<0.05 compared to slices incubated in septic serum, Scale bars= 20nm
